# UPLC-MS/MS for the Herb-Drug Interactions of Xiao-Ai-Ping Injection on Enasidenib in Rats Based on Pharmacokinetics

**DOI:** 10.1155/2021/6636266

**Published:** 2021-02-23

**Authors:** Bo-wen Wang, Cheng-zheng Qiu, Chang-qian Tang, Jia-hui Zhang, Yi Zhang, Qi-guang Du, Yi Feng, Xiang-jun Qiu

**Affiliations:** School of Basic Medical Sciences, Henan University of Science and Technology, Luoyang 471023, China

## Abstract

**Objective:**

To develop and validate a sensitive and rapid ultraperformance liquid chromatography-tandem mass spectrometry (UPLC-MS/MS) method for the determination of enasidenib in rat plasma and to investigate the effect of Xiao-ai-ping injection (XAPI) on the pharmacokinetics of enasidenib in rats.

**Methods:**

The rat plasma was precipitated with acetonitrile, enasidenib and internal standard (IS) were separated on an Acquity UPLC BEH C18 column, and acetonitrile and 0.1% formic acid were used as the mobile phase in gradient mode. Enasidenib and IS were monitored and detected by multiple reaction monitoring (MRM) using tandem mass spectrometry in positive ion mode. 12 Sprague-Dawley (SD) rats were randomly divided into control group (group A) and experimental group (group B), 6 rats in each group. Group B was intramuscularly injected with XAPI (0.3 mL/kg) every morning, 7 days in a row. Group A was intramuscularly injected with normal saline, 7 days in a row. On the seventh day, enasidenib (10 mg/kg) was given to both groups 30 min after injection of normal saline (group A) or XAPI (group B), and the blood was collected at different time points such as 0.33, 0.67, 1, 1.5, 2, 3, 4, 6, 9, 12, 24, and 48 h. The concentration of enasidenib was detected by UPLC-MS/MS, and the main parameters of pharmacokinetic of enasidenib were calculated using the DAS 2.0 software.

**Results:**

Under the current experimental conditions, this UPLC method showed good linearity in the detection of enasidenib. Interday and intraday precision did not exceed 10%, the range of accuracy values were from -1.43% to 2.76%. The results of matrix effect, extraction recovery, and stability met the requirements of FDA approval guidelines of bioanalytical method validation. The *C*_max_ of enasidenib in the group A and the group B was (458.87 ± 136.02) ng/mL and (661.47 ± 107.32) ng/mL, *t*_1/2_ was (7.74 ± 0.91) h and (8.64 ± 0.42) h, AUC_(0 − *t*)_ was (4067.24 ± 1214.36) ng·h/mL and (5645.40 ± 1046.30) ng·h/mL, AUC_(0 − ∞)_ was (4125.79 ± 1235.91) ng·h/mL and (5759.61 ± 1078.59) ng·h/mL, respectively. The *C*_max_ of enasidenib in group B was 44.15% higher than that in group A, and the AUC_(0 − *t*)_ and AUC_(0 − ∞)_ of enasidenib in group B were 38.80% and 39.60% higher than that in group A, respectively, and the *t*_1/2_ was prolonged from 7.74 h to 8.64 h.

**Conclusion:**

An UPLC-MS/MS method for the determination of enasidenib in rat plasma was established. XAPI can inhibit the metabolism of enasidenib and increase the concentration of enasidenib in rats. It is suggested that when XAPI was combined with enasidenib, the herb-drug interaction and adverse reactions should be paid attention to, and the dosage should be adjusted if necessary.

## 1. Introduction

Herb-drug interaction (HDIs) is a major health problem worldwide due to the potential risk of adverse reactions. The pharmacokinetic and pharmacodynamic changes resulting from this interaction may result in toxic or subtherapeutic outcomes associated with adverse clinical outcomes [1]. A large number of cancer patients worldwide use herbal supplements during cancer treatment, and the number is reported to be increasing. The risk of drug interactions is common, especially since the advent of oral anticancer drugs (OAA) (i.e., oral anticancer drugs such as hormone therapy, chemotherapy, and targeted therapy) [2].

Xiao-ai-ping injection (XAPI) is an injection extracted from Chinese herbal medicine *Marsdenia tenacissima*, and the main components of XAPI contain phenolic acids and steroidal glycosides, among other compounds [3, 4]. In recent years, pharmacological studies have shown that XAPI has the following functions: inhibiting tumor growth, preventing tumor cell invasion and metastasis, inducing tumor cell apoptosis, inhibiting tumor angiogenesis, and improving immunity. XAPI combined with chemotherapy drugs is a very useful treatment strategy, especially in combination with XELOX (capecitabine plus oxaliplatin) [5]. XAPI can promote the infiltration and function of CD8^+^ T cells, thus, enhancing the antigrowth effect of cisplatin on Lewis lung carcinoma (LLC) xenogeneic mice, which provides new evidence for the clinical application of XAPI and cisplatin [6]. XAPI combined with platinum chemotherapy has a better tumor response, improve the quality of life of patients, reduce adverse reactions, enhance immune function, and can be used in the treatment of advanced nonsmall cell lung cancer [7].

**Figure 1:**
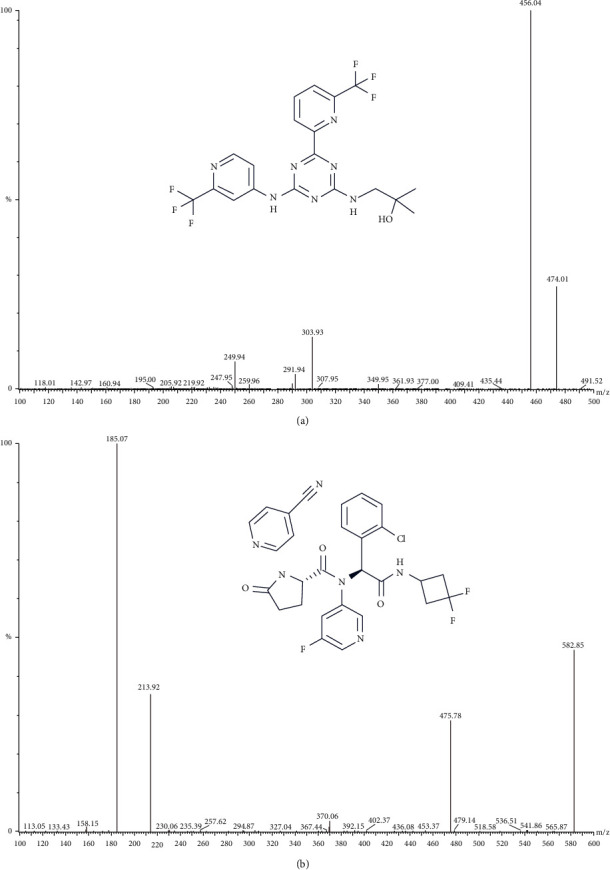
The ion transitions from parent ion to daughter ion and the chemical structure of enasidenib (a) and ivosidenib (b) in the present research.

Enasidenib (Idhifa®, Figure 1(a)) is an oral selectively allosteric inhibitor of isocitrate dehydrogenase 2 (IDH2) mutation. Enasidenib is developed by Celgene Corporation under a global exclusive license from Agios Pharmaceuticals. Enasidenib shows more powerful inhibitory effects on IDH2 R172K rather than IDH1 R140Q mutation, although it has inhibitory effects on both the mutations [8]. Enasidenib has been approved on 1 August 2017 for the treatment of adult relapsed/refractory acute myeloid leukemia (R/R AML) with IDH2 mutations. The recommended oral dose is 100 mg once daily for at least 6 months or until disease progression or intolerable adverse reaction [9]. Enasidenib is an oral, small molecule agent, currently approved to be taken at a dose of 100 mg daily [10]. Enasidenib is metabolized via CYP and UGT enzymes [8], including CYP1A2, CYP2B6, CYP2C8, CYP2C9, CYP2C19, CYP2D6, CYP3A4 and UGT1A1, UGT1A3, UGT1A4, UGT1A9, UGT2B7, and UGT2B15 [11]. In vitro studies suggest enasidenib inhibits multiple hepatic and gastrointestinal enzymes and drug transport proteins, including CYP1A2, CYP2B6, CYP2C8, CYP2C9, CYP2C19, CYP2D6, CYP3A4, UGT1A1, P-gp, BCRP, OAT1, OATP1B1, OATP1B3, and OCT2 [11]. At the same time, It can notably induce enzymes CYP3A4 and CYP2B6 [8]. Because enasidenib may induce or inhibit drug-metabolizing enzymes and transporters, combination therapy can increase or decrease the combination concentration [12].

There is also growing interest in the use of complementary and alternative medicine (CAM). 30% to 70% of patients with cancer have used CAM. In the daily management of cancer patients, herb-drug interactions (HDIs) should not be ignored by healthcare providers [13]. By contrast, surveys revealed that about one-third of patients using herbal supplements during their chemotherapy were at potential risk of HDIs. Mechanistically, the majority of potential HDIs occur on a pharmacokinetic level [14].

In recent years, the antitumor effect of XAPI has been further confirmed, and it has been widely used in the treatment of gastric cancer, lung cancer, esophageal cancer, and other malignant tumors. In the treatment of advanced gastric cancer, XAPI combined with chemotherapy is commonly used. Studies have shown that the extract of XAPI can interfere with the activities of CYP3A4 and CYP2D6 [15]. So, it may cause the HDIs based on CYP450.

Therefore, according to the previous research on the methodology of detecting enasidenib by ultraperformance liquid chromatography-tandem mass spectrometry (UPLC-MS/MS) [16], in this study, a sensitive, rapid, and simple method for determination of enasidenib in rat plasma by UPLC-MS/MS was developed and performed using ivosidenib as the internal standard (IS, Figure 1(b)), and the effect of XAPI on the changes of pharmacokinetics of enasidenib in rats was observed.

## 2. Materials and Methods

### 2.1. Chemicals

Enasidenib (purity above 98%) was purchased from the Beijing sunflower Technology Development Co., Ltd. Ivosidenib (purity over 98%) was purchased from sigma Aldrich (Shanghai) Trading Co., Ltd. HPLC grade formic acid was purchased from anaqua chemicals, Wilmington, USA. Acetonitrile and methanol of HPLC level were obtained from Merck Company (Darmstadt, Germany). Purified water was produced by utilizing a Milli-Q academic reagent grade water purification system (Millipore, Bedford, USA).

XAPI (batch number: National Pharmaceutical Standard Z20025868, 2 mL/piece) was purchased from Nanjing Shenghe Pharmaceutical Co., Ltd. (Nanjing, China).

### 2.2. Solutions Ready

Accurately weigh enasidenib and ivosidenib in different volumetric flasks, dissolve them with methanol, and prepare enasidenib and ivosidenib stock solution with the concentration of 1.0 mg/ml, respectively. Then, the stock solution was diluted with methanol to obtain various working solutions for calibration curve and quality control (QC) of enasidenib. The final concentrations of the calibration curves of enasidenib were as follows: 1, 5, 10, 50, 100, 250, 500, and 1 000 ng/mL. As for QC samples, they were made in the same way at three concentration levels (low, medium, and high concentration), and the concentrations of QC samples in plasma were 2.5, 250, and 750 ng/mL. The IS working solution (500 ng/mL) was also prepared by diluting the stock solution of ivosidenib with acetonitrile. All solutions were stored in a refrigerator at -20°C.

### 2.3. UPLC-MS/MS Conditions

The chromatographic column was Acquity BEH C18 column (2.1 mm × 100 mm, 1.7 *μ*m), and the temperature was set at 45°C. The mobile phase consisted of 0.1% formic acid in acetonitrile (A) and 0.1% aqueous formic acid (B), and the scheme was implemented as follows: 0-1.4 min (30%⟶85% A) and 1.4-2.0 min (85%⟶30% B). The flow rate was set at 0.40 mL/min, and the total run time was 2.0 min.

Mass spectrometry was conducted on a XEVO TQ-S triple quadrupole mass spectrometer equipped with a positive ion electrospray ionization (ESI) interface. Multiple reaction monitoring (MRM) mode was used to measure enasidenib with transitions of *m*/*z*474.02⟶456.01 for quantification, and *m*/*z*582.85⟶185.07 for quantification of IS, respectively. Maslynx 4.1 software (Milford waters, Massachusetts, USA) was used to realize the control and data acquisition of the instrument.

### 2.4. Sample Preparation

Accurately suck 50 *μ*L rat plasma into 1.5 mL EP tube, add 10 *μ*L of ivosidenib IS working solution (500 ng/mL), vortexed for 15 s, and add 200 *μ*L of acetonitrile. After vortex mixing for 1.0 min, centrifugation was performed at 10000 rpm for 15 min. 2 *μ*L of supernatant was taken and detected in UPLC-MS/MS system.

### 2.5. Method Verification

Validation is the basis of biological sample analysis. All the pharmacokinetic results depend on the analysis of biological samples. Only reliable methods can get reliable results. The established method should be verified through the study of accuracy, precision, specificity, sensitivity, recovery, stability, etc. According to technical guidelines for nonclinical pharmacokinetics of drugs of CFDA (China Food and Drug Administration) and the principles of Industry Bioanalytical Method Validation proposed by FDA, the method was validated [17, 18].

The analytical method should be able to distinguish the target analyte from the endogenous components of the IS and matrix or other components in the sample. Blank plasma samples from six different rats were used to demonstrate selectivity, which is generally acceptable when the response of the interfering component is less than 20% of the response to the lower limit of quantitation (LLOQ) of the analyte.

The response of the instrument to the analyte should be evaluated within the specified concentration range to obtain the standard curve. The final concentrations of the calibration curves of enasidenib were as follows: 1, 5, 10, 50, 100, 250, 500, and 1 000 ng/mL. Taking the peak area ratio of enasidenib to IS as ordinate (*y*) and theoretical concentration of enasidenib as abscissa (*x*), the standard curve was drawn with the least square method, and the weight factor was 1/*x*^2^. The LLOQ was regarded as the minimum value of the calibration curve when the value of S/N is more than 10, with accuracy (relative error, RE, %) at ±20% and precision (relative standard deviation, RSD, %) below 20%.

In a single analysis run, six repetitive measurements were used to calculate the precision and accuracy within a day at three QC concentrations (2.5, 250, and 750 ng/mL). Eighteen repeated measurements, at three separate days, were used to calculate interday precision and accuracy at three QC concentrations (2.5, 250, and 750 ng/mL). RSD% represents the precision while RE% expresses the accuracy.

For matrix effect, the ratio of peak area in the presence of matrix (measured by adding enasidenib and IS after extraction from blank matrix) to corresponding peak area without matrix (pure solution of enasidenib and IS) should be calculated. The calculation of extraction recovery was analyzed through dividing each analyte response spiked to drug-free plasma prior to extraction by the concentration after extraction. The extraction recovery as well as the matrix effect was determined in six replicates at three QC concentrations (2.5, 250, and 750 ng/mL).

The stability of three QC concentrations (2.5, 250, and 750 ng/mL) under four different conditions was studied. They were samples with three freeze-thaw cycles, samples at 4°C for 4 h in automatic sampler, samples at room temperature for 2 h, and samples stored at -20°C for 4 weeks. In this study, the stability of enasidenib in rat plasma was evaluated by analyzing five plasma samples with different concentrations.

### 2.6. Animals

Twelve SD rats were provided by the Laboratory Animal Center of Henan University of Science and Technology, and the animal production license number was SCXK (Hubei) 2010-0007. All experimental animals were authorized by the Ethics Committee of Medical Department of Henan University of science and technology and used on the basis of experimental animals. The experiment was approved according to the Laboratory animals—guidelines for ethical review of welfare (GB/T 35892-2018). The animal feeding conditions were as follows, temperature of 16~28°C, relative humidity of 40% ~70%, and feeding twice a day. One day before the experiment, all rats stopped feeding and were free to drink water.

### 2.7. Experiment Design

Twelve SD rats were randomly divided into control group (group A) and experimental group (group B), 6 rats in each group. Group B was intramuscularly injected with XAPI (0.3 mL/kg) every morning, 7 days in a row. Group A was intramuscularly injected with normal saline, 7 days in a row. On the seventh day, enasidenib (10 mg/kg) was given to both groups 30 min after injection of normal saline (group A) or XAPI (group B), and the blood was collected from the tail vein of each rat and put into 1.5 mL heparinized tubes, and at different time points such as 0.33, 0.67, 1, 1.5, 2, 3, 4, 6, 9, 12, 24, and 48 h. The blood samples were collected into polyethylene tubes containing heparin; the plasma was taken and frozen at -20°C until analysis.

### 2.8. Plasma Sample Detection

The concentration of enasidenib in rat plasma of group A and group B was detected by the abovedeveloped UPLC-MS/MS method, according to the method of batch analysis. Blank rat plasma was used as the matrix to prepare the plasma standard curve and QC samples. Prepare the accompanying standard curve and measure the quality control sample to ensure the reliability of the biological sample analysis data.

### 2.9. Data Analysis

The DAS 2.0 software was used to calculate the main pharmacokinetic parameters of enasidenib with the noncompartmental analysis. The data were expressed as mean ± standard deviation (SD). The data were processed using SPSS 18.0 statistical software. *P* values were calculated using the Independent sample *t*-test, and *P* < 0.05 was considered statistically significant.

## 3. Result

### 3.1. Specificity

**Figure 2:**
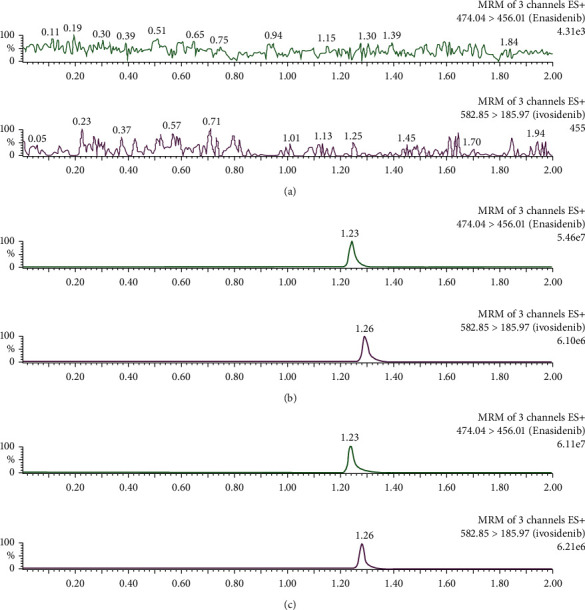
The representative chromatograms of enasidenib and IS. A blank plasma sample (a), a blank plasma sample spiked with 500 ng/ml enasidenib and IS (b), and a rat plasma sample 1.0 h after oral administration of 10 mg/kg enasidenib (c).

Under the current experimental conditions, enasidenib and IS could be well separated. The representative chromatograms were shown in Figure 2. A represents a blank plasma sample, B represents a blank plasma sample spiked with enasidenib and IS, and C represents a rat sample. The mean retention times of enasidenib and IS were 1.23 and 1.26 min, respectively.

### 3.2. Linearity

In the range of 1–1 000 ng/mL of the plasma concentration of enasidenib, the typical regression equations of enasidenib were *y* = 1.5 × 10^−3^ *x* − 1.34 × 10^−2^ (*R*^2^ = 0.999 3). *y* represents the peak area ratio (enasidenib peak area/IS peak area), and *x* represents the plasma concentration of enasidenib. The LLOQ of enasidenib was the minimum concentration of the standard curve (1 ng/mL).

### 3.3. Precision and Accuracy

The results obtained for the intraday precision and interday precision of enasidenib were shown in Table 1. The precision (% RSD) for enasidenib did not exceed 8.07%. The accuracy (% RE) for enasidenib was in the range from -1.20% to 2.76%. The results of precision and accuracy meet the requirements of methodology validation.

### 3.4. Recovery and ME

The recovery and ME results were investigated and shown in Table 2. The recoveries of enasidenib in rat plasma were higher than 80%, and the matrix effect met the requirements of detection.

### 3.5. Stability

All results for the stability samples test are summarized in Table 3, and they were within the acceptable criteria of ±15%, indicating that enasidenib was stable under the four conditions described.

### 3.6. The Effect of XAPI on the Pharmacikinetics of ENA

**Figure 3:**
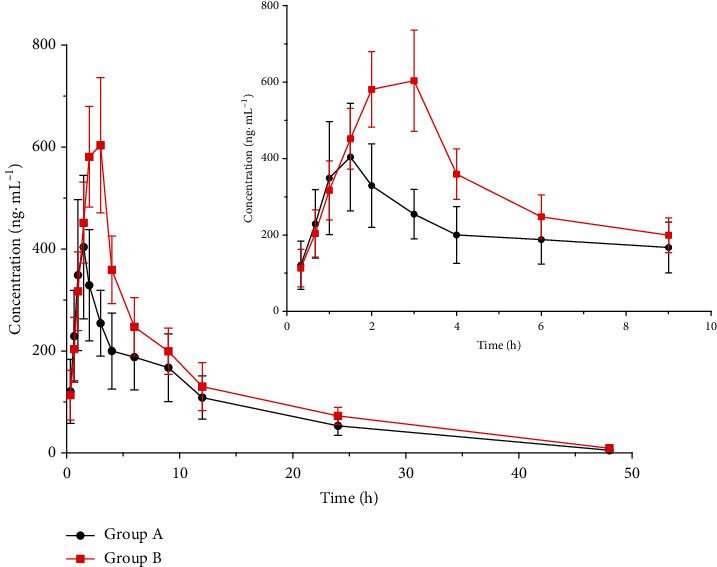
Plasma mean concentration-time curve of enasidenib (zoomed 1 to 9 h pharmacokinetic profile).

The plasma concentration-time curve of enasidenib was shown in Figure 3, and the main pharmacokinetic parameters of enasidenib were shown in Table 4. The *C*_max_ of enasidenib in group B was 44.15% higher than that in group A, and the AUC_(0 − *t*)_ and AUC_(0 − ∞)_ of enasidenib in group B were 38.80% and 39.60% higher than that in group A, respectively, and the *t*_1/2_ was prolonged from 7.74 h to 8.64 h. At the same time, CL was smaller and MRT was longer in group B than that in group A. The results showed that XAPI could slow down the metabolism of enasidenib and increase the plasma drug concentration of enasidenib in rats.

## 4. Discussion

The UPLCMS/MS method for the determination of enasidenib in rat plasma using ivosidenib as the internal standard has high sensitivity, short analysis time, and simple sample operation. In order to obtain a shorter analysis time and more symmetrical peaks, we optimized the chromatographic conditions, including chromatographic column and mobile phase. The Acquity UPLC CSH C18 column, BEH C18 column, and HSS C18 column were evaluated. The results showed that enasidenib and IS had good separation on the UPLC BEH C18 column. We investigated the mobile phase ratio of acetonitrile, methanol, formic acid, and acetic acid under different conditions. The results showed that when acetonitrile and 0.1% formic acid were used as the mobile phase, the chromatographic peak was the best.

Multiple reaction monitoring (MRM) was a commonly used analytical method for mass detection. It has the outstanding advantages of strong specificity, high sensitivity, high accuracy, good reproducibility, wide linear dynamic range, and high-throughput automation [15]. At the same time, we optimized the mass spectrum conditions, including the most sensitive ionization mode. The results show that the sensitivity is the highest under the condition of positive ion monitoring.

The incidence of herb reactions affected by TCM preparations may be more frequent than expected clinically because patients are more likely to self-administer without informing their healthcare providers. Some interactions may have beneficial effects by improving drug efficacy or reducing potential side effects. However, more often than not, the combination of herbal products and traditional medicines may bring about potential side effects and outcome effects, which are always difficult to predict [19].

Chinese medicine as an important means of cancer treatment in China has a history of many years. Traditional Chinese medicine (TCM) can inhibit the growth of tumor and prolong the survival rate of cancer patients [20]. But herb-drug interactions are a major health problem due to the risk of potential adverse reactions. Pharmacokinetic and pharmacodynamic variations derived from this type of interaction can produce toxicity or subtherapeutic results associated with undesirable clinical consequences [1], and the majority of potential herb-drug interactions occur on a pharmacokinetic level [14].

Enasidenib is a new and first-class oral small molecule inhibitor of IHD2. It is a noncytotoxic differentiation agent, which plays a role in inducing malignant cells to mature [12]. Enasidenib is metabolized via CYP and UGT enzymes [8]. Studies have shown that the extract of XAPI can interfere with the activities of CYP3A4 and CYP2D6 [15]. So, it may cause the herb-drug interactions based on CYP450. The results of this study showed that enasidenib was used in combination with XAPI. The *C*_max_ of enasidenib in group B was 44.15% higher than that in group A, and the AUC_(0 − *t*)_ and AUC_(0 − ∞)_ of enasidenib in group B were 38.80% and 39.60% higher than that in group A, respectively, and the *t*_1/2_ was prolonged from 7.74 h to 8.64 h. CL was smaller and MRT was longer in the experiment group than that in the control group. The results showed that XAPI could slow down the metabolism of enasidenib and increase the plasma concentration of enasidenib in rats. Therefore, when XAPI and enasidenib should be combined clinically, the dosage should be adjusted to ensure the efficacy to avoid adverse reactions.

## 5. Conclusions

A method for the determination of enasidenib in rat plasma was established. XAPI can inhibit the metabolism of enasidenib and increase the concentration of enasidenib in rats. It is suggested that when XAPI is combined with enasidenib, the herb-drug interaction and adverse reactions should be paid attention to, and the dosage should be adjusted if necessary.

## Figures and Tables

**Table 1:** The precisions of enasidenib in rat plasma (*n* = 6, mean ± SD).

Added (ng/mL)	Intraday			Interday		
	Found (ng/mL)	RSD (%)	RE (%)	Found (ng/mL)	RSD (%)	RE (%)
1	0.98 ± 0.41	4.16	-1.61	0.98 ± 0.35	3.54	-2.52
2.5	2.47 ± 0.16	6.28	-1.20	2.48 ± 0.14	5.52	-0.80
250	251.75 ± 12.84	5.10	0.70	249.17 ± 13.16	5.28	-0.33
750	741.62 ± 16.69	2.25	-1.12	752.16 ± 17.06	2.27	0.58

**Table 2:** The recoveries and ME of enasidenib in rat plasma (*n* = 6, mean ± SD).

Added (ng/ml)	Recovery (%)	RSD (%)	ME (%)	RSD (%)
2.5	80.69 ± 3.18	3.94	102.07 ± 5.46	5.35
250	82.08 ± 2.83	3.44	99.60 ± 6.05	6.07
750	83.97 ± 2.60	3.09	100.85 ± 2.51	2.49

**Table 3:** The stability of enasidenib in rat plasma (*n* = 6, mean ± SD).

Added (ng/mL)	Room temperature, 2 h		Autosampler 4 °C, 4 h		Three freeze-thaw		-20°C, 4 weeks	
	RSD (%)	RE (%)	RSD (%)	RE (%)	RSD (%)	RE (%)	RSD (%)	RE (%)
2.5	7.01	-3.53	6.45	1.35	6.02	-1.00	4.45	1.07
250	7.07	2.26	5.38	-1.59	5.44	1.85	5.18	-0.55
750	1.99	-0.42	2.54	0.33	1.63	0.18	1.26	-0.31

**Table 4:** The main pharmacokinetic parameters of enasidenib (*n* = 6, mean ± SD).

Parameters	Group A	Group B
*t*_1/2_ (h)	7.74 ± 0.91	8.64 ± 0.42
*T*_max_ (h)	1.33 ± 0.41	2.50 ± 0.55^∗∗^
*C*_max_ (ng/mL)	458.87 ± 136.02	661.47 ± 107.32^∗^
CL_*z*_/*F* (L/h/kg)	2.70 ± 1.13	1.79 ± 0.35^∗∗^
*V*_*z*_/*F* (L/h)	30.52 ± 15.33	22.23 ± 3.88^∗^
MRT_(0 − *t*)_ (h)	11.10 ± 1.03	11.01 ± 0.88
MRT_(0 − ∞)_ (h/)	11.78 ± 0.99	11.97 ± 1.07^∗^
AUC_(0 − *t*)_ (ng·h/mL)	4067.24 ± 1214.36	5645.40 ± 1046.30^∗^
AUC_(0 − ∞)_ (ng·h/mL)	4125.79 ± 1235.91	5759.61 ± 1078.59

Abbreviations: *t*_1/2_: half-life; *T*_max_: time of peak concentration; MRT_(0 − *t*)_: mean residence time of 0 − *t* time; MRT_(0 − −∞)_: mean residence time of 0-infinity time; *C*_max_: peak concentration; AUC_(0 − *t*)_: area under curve of 0 − *t* time; AUC_(0 − ∞)_: area under curve of 0-infinity time. (Compared with the Group A, ^∗^*P* < 0.05; ^∗∗^*P* < 0.01).

## Data Availability

The data used to support the findings of this study are available from the corresponding author upon request.
